# Opening up? Exploring motives and needs of students and staff of a Dutch university on disclosing mental health issues to inform decision aid development

**DOI:** 10.1371/journal.pone.0333042

**Published:** 2025-11-03

**Authors:** Yil Engbersen-Severijns, Daniëlle N. Zijlstra, Sanne E. M. Brouwers, Femke M. den Uil, Véronique Vancauwenbergh, Thomas Gültzow

**Affiliations:** 1 Department of Health Psychology, Open University of the Netherlands, Heerlen, the Netherlands; 2 Department of Health Promotion, Maastricht University, Maastricht, the Netherlands; 3 Department of Work and Social Psychology, Faculty of Psychology and Neuroscience, Maastricht University, Maastricht, the Netherlands; 4 Student Services Centre, Department Personal and Professional Development, Maastricht University, Maastricht, the Netherlands; 5 Department of Theory, Methods and Statistics, Open University of the Netherlands, Heerlen, the Netherlands; School of Nursing Sao Joao de Deus, Evora University, PORTUGAL

## Abstract

**Background:**

Decisions about disclosing mental health issues can be difficult, especially in the university setting. For this reason, students and staff of universities might benefit from decision support tools, such as the use of a decision aid. Currently, little is known about if and how people in this setting disclose and how decision support should look like. This study aims to address this gap by exploring disclosure experiences and preferences for support.

**Methods:**

Semi-structured interviews were conducted with staff (n = 10) and students (n = 10), including participants who decided to (not) disclose their mental health condition. The participants were asked to complete a short questionnaire. Subsequently, they were interviewed about the decision to disclose and their needs regarding a potential disclosure decision aid. Interviews were analyzed using the Framework method.

**Results:**

Most students chose not to disclose, whereas most staff did. Students and staff described advantages and disadvantages of disclosing. Reported advantages included support, relief, understanding, and safety. Risks included (fear of) stigmatization and career concerns. Not disclosing can prevent unwanted questions but can also lead to misunderstandings. Regarding decision support, both students and staff preferred a brief online tool.

**Conclusion:**

In conclusion, both students and staff encounter challenges when it comes to deciding whether to disclose their mental health status. A decision aid can be a valuable tool to support them in making their decision.

## Introduction

In the Netherlands, more than four out of 10 people experience mental health issues in their lives at some point [1,2]. These issues refer to different mental health conditions that can affect someone’s mood, thinking, behavior, and daily life and can be either chronic or temporary [3,4]. While mental health issues are widespread, certain population groups, including but not limited to students and university staff, find themselves in an environment that may both facilitate the development and concealment of these issues. For example, it is often reported nationally as well as internationally that students consistently report higher levels of mental health issues than the general population [5–8]. Furthermore, it has been reported that they often disclose their issues to university staff that may not be trained to be mental health professionals which in turn could hinder disclosure in the first place [9]. At the same time, university staff also shows specific types of mental health issues, such as burnout, at high rates similar to other service sector employees, such as school teachers and healthcare professionals [10]. Furthermore, a survey conducted in the Netherlands revealed that 39% of PhD candidates, representing one category of university staff, exhibited severe burnout symptoms, and 47% were found to be at risk of developing psychiatric disorders. However, this issue is not confined to the Netherlands alone, as global statistics indicate that 36% of PhD candidates worldwide have sought assistance for mental health challenges stemming from their doctoral trajectories [11]. At the same time, it is known that people often tend to find it more difficult to disclose mental health issues in the workplace than in social contexts [12].

While having mental health issues in itself can have negative effects on study progress, work performance, and private life, deciding not to disclose might further impede support efforts offered by other people [13]. However, students and staff affected by mental health issues may doubt whether to inform other people about their mental health issues due to several reasons. For example, both students and staff of universities may fear to be stigmatized if they disclose their problems to others [12,14]. Also, disclosing one’s mental health issues can lead to problems when entering the labor market. To exemplify this, a recent Dutch survey has shown that 64% of Dutch line managers are reluctant to hire people with mental illnesses [15]. Therefore, it is important to minimize mental health stigma among people [16], especially as the decision to disclose mental health issues can help to access and receive adequate support. Other research has, for example, shown that disclosing mental health issues can increase individual well-being and reduce self-stigma [13]. This complex interplay of advantages and disadvantages associated with disclosure underlines the importance of carefully considering the decision to disclose. However, at the same time, we know that people can experience (deliberative) decision making as difficult in other health domains [17]. That said, to our knowledge, it has never been investigated how Dutch university staff and students experience the decision-making process surrounding mental health disclosure decisions. Therefore, our first research question (RQ) is:

1. What are the motives, considerations, and needs of students and staff of a Dutch University (i.e., Maastricht University) regarding the decision to disclose their mental health issues (or not)?

Although it is currently unclear how Dutch university staff and students experience this decision-making process, it is known from other health domains that individuals can benefit greatly from the use of interventions intended to support informed decision-making processes, so-called decision aids [18]. To expedite the development of a mental health disclosure decision aid, we aimed to not only explore the current experience of the decision-making process but also examined the needs and opinions of students and staff of Maastricht University to enable later development of a mental health disclosure decision aid. Our second RQ therefore is:

1. What are needs and opinions of students and staff of a Dutch University (i.e., Maastricht University) regarding a decision aid aimed at supporting them in deciding to disclose their mental health issues (or not)?

To answer both RQs we have conducted interviews to gain insights in the decision-making process and the support needs of students and staff of Maastricht University. The end goal is to develop a decision aid which can support staff and students at Maastricht University in their decision whether they want to disclose their mental health issues. With this decision aid, we aim to support safe and informed mental health disclosure decisions, while also reducing the stigma around mental health issues.

This article describes the results of a qualitative study conducted among students and staff of Maastricht University.

## Materials and methods

A qualitative study was conducted using semi-structured interviews with students and staff of Maastricht University, as one example of a Dutch university. Maastricht University is characterized as one of the youngest and also one of the most international universities in the Netherlands. This research was conducted in accordance with the principles outlined in the Declaration of Helsinki and received approval from the Ethics Review Committee Psychology and Neuroscience (ERCPN) of Maastricht University (188_10_02_2018_S123) and has been preregistered on the Open Science Framework (https://osf.io/vwaer). This study adheres to the COREQ (Consolidated Criteria for Reporting Qualitative Research) checklist for the reporting of qualitative research (S3 File).

### Participants and recruitment

Participants were eligible for participation if they were either a staff member or a student at Maastricht University and had mental health issues, e.g., anxiety disorders, depression, loneliness, or obsessive-compulsive disorder; all participants happened to be 18 years or older, although age was not an inclusion criterion. We intentionally opted to also include participants without a formal diagnosis and have used the term mental health issues in a broader sense to also encompass the experiences of individuals who may not have sought formal mental health care (yet). Specifically, we used the following definition: “Mental health issues mean all psychological and emotional complaints that can prevent people from functioning optimally in daily life (e.g., anxiety or depression)”. Additionally, we purposefully also included both participants who chose to disclose as well as those who chose not to disclose. Participants were recruited via mailing lists within different departments of Maastricht University, via announcements for students on the online learning management system used by the university, WhatsApp, and LinkedIn (S1 File). The recruitment messages included information about the study’s rationale, eligibility criteria, and what participation would involve. Recruitment ran from 5 September 2022–5 October 2022. When people were interested, they could contact the research team via e-mail. After they contacted the research team, interview appointments were made by phone or e-mail. Participants could select their preferred interview format, either via Zoom or in-person at the university, and also had the choice of being interviewed by either a student assistant or a staff member. While none of the participants had a close personal or professional relationship with their interviewer, some participant–interviewer pairs were already acquainted prior to the interviews. If not, no prior contact was established before the interview took place (except for planning the interview itself) and no personal information (except whether the interviewer was a student or staff member) was disclosed. After the interview, participants received a gift card worth 10€.

### Interviews

Before the start of the interviews, participants were asked to read the patient information letter and provide written informed consent by checking a box in an online form. After this, participants were asked to fill out a brief questionnaire(English: https://osf.io/j9rsthttps://osf.io/j9rsthttps://osf.io/j9rst; Dutch: https://osf.io/m2983https://osf.io/m2983https://osf.io/m2983)  (English: https://osf.io/j9rst; Dutch: https://osf.io/m2983) containing questions related to demographic information, the difficulty of the decision-making process, and the SURE test [11]. The interviews were semi-structured by means of an interview guide that was developed a priori(English: https://osf.io/qcnsfhttps://osf.io/qcnsfhttps://osf.io/qcnsf; Dutch: https://osf.io/87z5jhttps://osf.io/87z5jhttps://osf.io/87z5j) (English: https://osf.io/qcnsf; Dutch: https://osf.io/87z5j) based on the Ottawa Decision Support Framework [19]. The interview guide was divided in two main parts: (1) motives, considerations, and needs of students and staff of Maastricht University regarding the decision to disclose their mental health issues (or not) and (2) needs and opinions regarding the development of the decision aid. The first topic was related to the mental health issues the interviewees experienced, how they made the decision to disclose or not and how they felt about making this decision. The second topic was related to the decision aid. The full interview guide can be found on the Open Science Framework(English: https://osf.io/qcnsfhttps://osf.io/qcnsfhttps://osf.io/qcnsf; Dutch: https://osf.io/87z5j).https://osf.io/87z5jhttps://osf.io/87z5j (English: https://osf.io/j9rst; Dutch: https://osf.io/m2983), it was not formally pilot tested. Two(SB and FdU students (SB, FdU, both women, close to finishing their BSc)) trained with little experience in qualitative research before this study and three staff members (TG, YS, DZ, 2 women, 1 men, all PhD) trained and experienced in qualitative(DZ (PhD), woman, TG (PhD), man and YS (PhD), woman research (*blinded) conducted the interviews. The interviews lasted between 30 and 60 minutes and only participants and the interviewers were present. Interviews were conducted until data saturation was achieved, defined as the point at which no new themes or insights emerged from the data. Saturation was considered reached when no new themes, categories or insights emerged from subsequent interviews, indicating that additional data collection would likely not add further value to the study findings but would only place an unnecessary burden on participants [20].

### Data analysis

All interviews were audiotaped, transcribed, and analyzed. Transcripts were not returned to participants for member checking, as the researchers aimed to minimize participant burden and deemed the likelihood of participants substantially altering their original views to be low. Analyses were done by using ATLAS.ti and using the Framework method (Gale et al., 2013). The transcription was conducted by five members of the research(SB, TG, YS, FdU, and DZ).  team (SB, TG, YS, FdU and DZ). After the transcription,SB, TG, and YS  SB, TG, and YS developed a coding tree. To ensure reliability, three members of the research team(TG, SB, and FdU)  TG, SB, and FdU first applied this coding tree to only one interview, instead of two as preregistered, because we determined that this one interview provided sufficient data for our purposes at the time. Minor changes were made to the coding tree where necessary andSB, TG, YS, FdU, and DZ SB, TG, Fdu, and DZ approved the final version used.TG, SB, and FdU Subsequently, TG, SB and FdU proceeded to code a pair of the same two interviews within ATLAS.ti, to compute both percentage agreement (as originally preregistered) and Krippendorff’s alpha. This choice of Krippendorff’s alpha was made since ATLAS.ti does not support Cohen’s Kappa, which was the measure we originally preregistered. Initially, we coded without prespecified segments, however after multiple rounds of coding we still had not achieved sufficient scores for both percentage agreement and Krippendorffs’ alpha, we decided to work with prespecified segments that were selected by TG. With these prespecified segmentsTG, SB, and FdU TG, SB, and FdU achieved a percentage agreement of 92.3% and Krippendorff’s alpha of 0.93. We regarded both as sufficient, also based on the preregistered cut-off of 90% for percentage agreement. Finally, we used the Framework Method to analyze the data, a flexible and pragmatic approach to qualitative data analysis that is not bound to a single epistemological stance. YS analyzed the coded data using framework matrixes. The analyses for this were based on the codes directly related to the research questions and the themes followed these codes. The questionnaires were analyzed descriptively in SPSS (version 28.0).

## Results

The results will be divided in two parts (1) the motives, considerations and needs of students and staff of Maastricht University regarding the decision to disclose their mental health issues and (2) their needs and opinions regarding the development of the decision aid.

### Characteristics of participants

In total, 20 participants (*n* = 10 staff, *n* = 10 students) were included. Eight staff members and one student chose to disclose their mental health issues within the university before the interviews took place. Participants experienced different mental health issues. Among the staff members, anxiety was the most common, whereas among the students, depression was the most prevalent issue. In total, five staff members and five students found it (very) difficult to make the decision, while three staff members and one student found it easy to make this decision. More information can be found in Table 1.

**Table 1:** Participants’ characteristics.

**Gender (*n*)**	
*Staff*	
Men	3
Women	6
Non-binary	1
*Students*	
Men	0
Women	10
Non-binary	0
**Age (*mean*)**	
Staff	33.9 (7.71)
Students	21.8 (1.99)
**Disclosure prior to the interview (*n*)**	
Staff	8
Students	1
**Non-disclosure prior to the interview (*n*)**	
Staff	2
Students	9
**Disclosure difficulty (*n*)**	
*Staff*	
Very difficult	1
Difficult	4
Neither difficult nor easy	2
Easy	3
Very easy	0
*Students*	
Very difficult	0
Difficult	5
Neither difficult nor easy	4
Easy	1
Very easy	0

*Note.* We chose not to report individual mental health issues to prevent privacy issues from occurring.

The SURE test results revealed that most staff members (*n* = 6) and some students (*n* = 5) were confident in their optimal choices. Meanwhile, some participants, including both staff (*n* = 4) and students (*n* = 5), were unaware of the benefits and risks of (not-)disclosing. However, most (staff *n* = 7, students *n* = 7) were aware of the specific benefits and risks that mattered most to them. Only some participants (staff *n* = 3, students *n* = 4) indicated to lack sufficient support and guidance to make a decision. More information can be found in Table 2.

**Table 2:** SURE test.

	Staff (n)		Students (n)	
	*Yes*	*No*	*Yes*	*No*
**Do you feel SURE about the best choice for you?**	6	4	5	5
**Do you know the benefits and risks of each option?**	6	4	5	5
**Are you clear about which benefits and risks matter most to you?**	7	3	7	3
**Do you have enough support and advice to make a choice?**	7	3	6	4

### Decisional experiences

Most participants indicated that the decision to disclose their mental health issues is not a one-time decision but is dependent on different people and times in their lives. Additionally, the decision and the decisional experience is influenced by contextual factors (e.g., relationship with supervisor). Given that our focus was on the decision aid development, we focus on the advantages and disadvantages of disclosing.

### Advantages and disadvantages of (not) disclosing mental health

In the following text we will describe a comprehensive overview of the perceived (dis)advantages of disclosing mental health. A full overview of framework matrixes including the (dis)advantages can be found in the tables of S2 File.

#### Students’ (dis)advantages of disclosing.

Students mentioned different advantages of disclosing mental health issues related to practical as well as emotional aspects. On a more practical note, students mentioned that people can support them in finding solutions after disclosing. Relatedly, study adaptations, if possible, can only be made after disclosing. Emotional advantages of disclosing where that others can create safe spaces after disclosing, that disclosing can induce feelings of relief and being understood, while also providing a sense of not being alone. Related to this, disclosing can make it easier for others to talk about their mental health issues as well.

Next to these advantages students also mentioned disadvantages of disclosing mental health issues. A practical concern that was raised was the considerable amount of time and energy required to engage in discussions with others. Other emotional disadvantages raised were experiencing stigma, the feeling of not been taken seriously, the experience that others are uncertain in which way to help and feeling like a burden to others.

#### Students’ (dis)advantages of not disclosing.

Advantages of not disclosing were the expectation that other people will not act differently towards them (after the disclosure), that it will prevent unpleasant reactions, stigma, and unsolicited help. Students also mentioned maintaining their privacy and not experiencing any anxiety in relation to disclosing.

Disadvantages of not disclosing were potential misunderstanding regarding one’s actions or choices and that, by not disclosing, mental health issues might become worse or – at least – will not improve. Students further expressed that their mental health issues could impact their academic performance, and that non-disclosure could hinder others’ comprehension of the underlying reasons.

#### Staff (dis)advantages of disclosing.

Like students, staff also mentioned advantages that related to both practical and emotional aspects. Practically, staff members noted that disclosure can help someone obtain necessary work adjustments and allow others to support one in finding solutions. Relatedly, staff members also mentioned that after disclosure one no longer has to make excuses, when they cannot engage in certain work activities. Emotional advantages were also similar to the ones experienced by students, i.e., staff members also indicated that disclosing could induce feelings of relief and being understood and supported. Again, they also shared that disclosing could help by feeling less alone which in turn could lead to better relationships with colleagues. Disclosure can also result in more self-compassion, positive reactions from others, and can also make it easier for others to disclose their mental health issues.

Disadvantages that were mentioned were that one could experience stigma, and uniquely for staff members, that this could negatively affect their career. Related to this, other people might also think that someone who discloses their mental health issues is not competent at their job. In addition, some employees found it difficult to discuss their mental health issue due to the perceived disadvantages of feeling like they are complaining or being misunderstood by others. Again, like students, the possibility of post-disclosure support was often cited as an advantage, but it can also result in unsolicited help. Some employees also said that disclosure does not always lead to increased empathy or support from others.

#### Staff (dis)advantages of not disclosing.

Again, staff members’ advantages for not disclosing mental health issues were very similar to those mentioned by students, i.e., preventing them from experiencing stigma and others treating them differently and eliminating the chance of unwanted inquiries about their condition.

On the other hand, opting not to disclose one’s mental health issues can lead others to speculate about one’s ability to effectively perform their work since they are unaware of the actual reasons behind any potential challenges. In other words, there is a possibility that it may impact others’ perception of job performance, that colleagues might think that you are not capable of doing your work. However, it was also mentioned that if one chooses not to disclose their mental health issues, it is unlikely that changes or accommodations will be provided. Also, if individuals do not attend work-related events due to undisclosed mental health issues, others may perceive them as not being part of the team. Lastly, some also mentioned that disclosing in itself can be stressful, so not disclosing prevents experiencing stress.

### Preferences towards the decision aid

In the following text we will describe a comprehensive overview of the decision aid preferences. A full overview can be found in the framework matrixes (additional file 4 (students) and 5 (staff)).

#### Preferences towards content.

While students expressed different preferences concerning decision aid content, the majority expressed a desire for the following in the decision aid: (1) contact information for university resources assisting with mental health issues, (2) information on the (dis)advantages of (non-) disclosure, (3) experience stories, and (4) an interactive component such as a decision tree to aid in decision making. Less frequently mentioned were a chat-function or frequently asked questions (FAQs). Many participants emphasized the importance of being able to visit the website anonymously, highlighting the significance of privacy.

The staff mentioned the same content elements as mentioned by students. Furthermore, staff members emphasized the importance of reporting the potential consequences of discussing mental health issues with others. Additionally, they specifically mentioned to include information about how Human Resources (HR) professionals can help you and what their roles are.

#### Preferences towards form.

Regarding the form, an online tool, such as a website with concise texts was preferred by most students and staff. The reason for this was the desire to access necessary information without overwhelming details when dealing with mental health issues. Additionally, ease of use was highlighted. Some students also mentioned a preference for video-content, for example so that people who can help with mental health issues at the university can introduce themselves. Both groups mentioned that (dis)advantages could be displayed in either a list or in text format, while experience stories for could be provided in text form, potentially accompanied by pictures or videos.

#### Strategies to inform potential users about the decision aid.

Staff members and students shared a plethora of strategies to inform potential users about the decision aid, with some stressing that multiple strategies will probably be needed. Students indicated that it would be good to be informed by teachers during mandatory classes/lectures, but also via (1) university e-mails, (2) posters, (3) brochures, (4) social media, (5) WhatsApp groups, and (6) the Diversity & Inclusivity Office of the university. Students also indicated that information could be spread via mandatory courses. Staff members mentioned that it would be good to be informed via (1) e-mail, (2) flyers, (3) supervisors, (4) newsletters, while also always directly informing new staff members and students about it.

#### When to use.

Most students and staff members preferred to use the decision aid at home when they have time to use it and when no other people are around. Some staff members mentioned that it would also be good to use the decision aid together with a professional from the university.

## Discussion

The aim of this study was to explore the decision-making process surrounding the disclosure of mental health issues among both students and staff at Maastricht University, as an example of a Dutch university. Additionally, we sought to explore their preferences regarding a decision aid for mental health disclosure.

In line with our recruitment strategy, which aimed to include participants who may or may not have disclosed their mental health issues, not all participants chose to disclose their mental health issues. Moreover, the interviews revealed an interplay of advantages and disadvantages that can lead to these differences in decision making. These advantages and disadvantages seemed to consist of both practical and emotional aspects. For example, disclosure can lead to receiving support and making necessary study or work-related adjustments, which are more practical benefits. On an emotional level, disclosure can provide relief and promote a sense of being understood, while also making it easier for others to disclose their mental health concerns. Disadvantages described were that disclosure can be time-consuming and emotionally taxing. In addition, many participants expressed concern about how others might react. Some were afraid of not being taken seriously, while others worried about stigmatization. Employees specifically indicated that this stigma could have a negative impact on their careers. In this regard our findings are in line with other publications that describe positive (e.g., increase of well-being [13]) and negative consequences of disclosure (e.g., discrimination and self-stigma [15,21]).

At the same time, these findings underline the complex nature of decision making around mental health disclosure, which in turn can lead to a state of uncertainty about the course of action to take regarding disclosure – often described in the scientific literature as decisional conflict [22–24]. Or in other words, the complex interplay of advantages and disadvantages could hinder people in the university context from deciding whether to disclose their mental health issues. That said, the results of the SURE test suggest that most participants in our sample do not encounter significant decisional conflict when it comes to deciding whether to disclose their mental health issues. However, it is important to consider that decision conflict typically diminishes after a decision is made [17]. Therefore, this observation could be more indicative of our recruitment approach, as we primarily enrolled individuals who had already made a decision, rather than providing a comprehensive overview of the entire university population. Providing mental health decision aids to students and staff before they make a decision could therefore still be useful, as these tools are known for their ability to assist individuals when faced with complex decisions involving involve a delicate balance between advantages and disadvantages [18].

Regarding decision aid preferences, participants mentioned that such a mental health disclosure decision aid should be an online tool that is easy to use and allows for anonymous use. Participants prefer an online tool as this will enable them to use it where and whenever they want it to use it. Most participants, compromising both students and staff, mentioned that such a decision aid should include contact information for university resources assisting with mental health issues, information on the advantages and disadvantages of (non)-disclosure, and personal narratives. Also, interactive elements were mentioned such as a decision tree that can guide users in their decision-making process. Additionally, staff members mentioned the importance of reporting potential consequences of disclosing mental health issues. These findings can be used to design mental health disclosure decision aids, especially those for the (Dutch) university setting, in fact, we have developed one for Maastricht University [25]. The insights gathered from the interviews have played a crucial role in shaping the content and design of the decision aid we have developed. For example, the expressed preference for interactive elements and the inclusion of practical information about potential consequences have been directly incorporated to enhance user engagement and relevance. This user-centered approach ensures that the decision aid reflects the lived experiences and concerns of its intended audience, thus increasing its acceptability and potential effectiveness. It should however be noted, that while the input from potential users is crucial in decision aid development [26], integration of their wishes should be carefully implemented using scientific evidence as well [27]. For example, in our sample participants expressed a desire for personal narratives within the decision aid. However, it is worth noting that such narratives are often viewed critically in the decision aid literature for their potential to influence users’ decisions [28]. Nevertheless, narratives that focus on describing processes and experiences rather than outcomes could make a positive contribution by promoting engagement, providing information and comfort without influencing the final decision [28]. In other words, decision aid developers should strive to integrate our findings with other scientific literature on decision aid development when developing mental health disclosure decision aids to ensure optimal effectiveness.

Ultimately, decision aids can help people to think carefully about their decision to disclose their mental health issues. Planning to disclose and strategically used decisional strategies can help to decrease the negative effects of stigma [29,30]. For example, when people decide to strategically disclose their mental health issues, it might be easier for them to have contact and peer association. Having contact with people who are recovering from their mental health issues, can help to reduce self-stigma [29]. Furthermore, a prior research, including a randomized controlled trial, has demonstrated that decision aids can help to reduce decisional conflict [31].

However, the findings also emphasize that disclosure decisions are embedded within broader institutional and cultural contexts. Staff members’ concerns about career-related stigma point to systemic barriers that cannot be resolved solely through individual decision aids. Therefore, while decision aids can empower individuals, their successful implementation should be part of a broader institutional commitment to reducing stigma and fostering safe environments. Future research and practice should consider how decision support tools may interact with organizational policies and culture to maximize their impact.

### Strengths and limitations

Although we largely achieved data saturation (i.e., the stage at which collecting additional data on a theoretical concept no longer reveals new characteristics, [32]) within our sample, the sample size imposes limitations. For instance, a few participants noted that their marginalized identities influenced their decision to disclose. Unfortunately, our sample size hindered a more in-depth exploration of this aspect. Consequently, future research should prioritize the inclusion of a diverse participant pool to thoroughly examine how intersecting identities might impact decisions related to mental health disclosure. Additionally, it is important to note that our interviews were conducted exclusively at one university situated in the Netherlands, which might hamper the generalizability of our findings. That said, they seem to be largely in line with the wider literature. Furthermore, it’s important to acknowledge the potential for gender bias in our findings, particularly within the student sample, due to the overrepresentation of women. However, this might also reflect the fact that it is known that more women in the Netherlands report mental health issues [33] and the negative effects of the Coronavirus disease 2019 (COVID-19) pandemic had a greater impact on the mental health of female students compared to male students [34]. It is worth highlighting that the staff sample displayed a somewhat more balanced representation of genders, including the inclusion of one non-binary participant. Another strength of this study in terms of representation is our inclusion of participants who both disclosed and did not disclose their mental health issues. However, as previously noted, future research could benefit from incorporating the perspectives of individuals currently in the process of deciding whether to disclose.

## Conclusion

In conclusion, our study shows that the decision to disclose mental health issues is complex and that this decision is influenced by several practical and emotional considerations. While some participants emphasized the benefits of disclosing such as receiving support, making adjustments or experience relief, others expressed concerns about stigma, emotional burden and even possible career implications. These experiences highlight that disclosure is often not a straightforward decision and often involves weighing perceived risks and benefits.

To support this decision-making process, participants expressed a need for a user-friendly, anonymous online decision aid. Preferred features included information on pros and cons of (non-)disclosure, links to university support services and interactive tools such as a decision tree. Based on these insights, we have developed an online decision aid for Maastricht University and plan to test the decision aid in the near future(https://osf.io/emwyu) (https://osf.io/emwyu). We also encourage other universities to prioritize mental health issues and mental health disclosure, as mental health issues and associated decisions affect many individuals.

## Supporting information

S1 File Example recruitment message. (DOCX)

S2 File Framework matrices. (DOCX)

S3 File COREQ checklist. (PDF)
