# Correction: Opening up? Exploring motives and needs of students and staff of a Dutch university on disclosing mental health issues to inform decision aid development

**DOI:** 10.1371/journal.pone.0341228

**Published:** 2026-01-20

**Authors:** Yil Engbersen-Severijns, Daniëlle N. Zijlstra, Sanne E. M. Brouwers, Femke M. den Uil, Véronique Vancauwenbergh, Thomas Gültzow

Another affiliation for the sixth author is missing. Thomas Gültzow is also affiliated with #3: Department of Work and Social Psychology, Faculty of Psychology and Neuroscience, Maastricht University, Maastricht, the Netherlands.

S3 File was uploaded incorrectly. Please see the correct S3 File here.

## Supporting information

S3 File COREQ checklist. (PDF)
