# Supplementary material for: Opening up? Exploring motives and needs of students and staff of a Dutch university on disclosing mental health issues to inform decision aid development
Source: PLoS One. 2025 Nov 3;20(11):e0333042. doi: 10.1371/journal.pone.0333042 (PMC12582453; doi:10.1371/journal.pone.0333042)
Supplement: S1 File — (DOCX) [file pone.0333042.s001.docx]

### **Supporting information**

### **S1 File. Example recruitment message**

Dear students and staff of (*blinded),

We need your help! We are looking for participants for our research.

We conduct research to gain insight in students and employees within Maastricht University who experience psychological problems, and who have chosen to ,whether or not,  talk about this with other people (e.g. other students/colleagues, psychologist, your mentor, study advisor) within the UM.

**Why are we doing this research?**

The aim of this research is to investigate which factors play a role in why people do or do not talk about psychological problems. By psychological problems, we mean all psychological and emotional complaints that can prevent you from functioning optimally in daily life (e.g. anxiety, depression). The aim is to develop a decision aid that can help people making the choice whether or not to talk about their psychological problems.

**Who can participate?**

·         Anyone who has been struggling with psychological problems in the past year/currently

·         Anyone who has chosen not to talk about this to someone within (*blinded)

·         Anyone who has chosen to talk about this to someone within (*blinded)

**What does participating mean?**

·         The research consists of an interview. This interview will last approximately **30 minutes**. The interview will be conducted by someone from the Opening-Up team.

·         In addition, we ask you to complete a very short questionnaire (**5 minutes**) prior to the interview.

·         The data of the interviews will be processed anonymously and cannot be traced back to you as a person.

·         You will receive **a gift card worth € 10**.

**How can I participate?**

If you would like to participate or have any other questions about the study, please contact:(*blinded)
