# Supplementary material for: Opening up? Exploring motives and needs of students and staff of a Dutch university on disclosing mental health issues to inform decision aid development
Source: PLoS One. 2025 Nov 3;20(11):e0333042. doi: 10.1371/journal.pone.0333042 (PMC12582453; doi:10.1371/journal.pone.0333042)
Supplement: S2 File — (DOCX) [file pone.0333042.s002.docx]

**S2. File. Framework matrices**

**Framework matrix students disclosing**

| Student | Disclosed at UM (to whom) and reason | Experience(s) | Advantages disclosing | Disadvantages disclosing | Advantages not disclosing | Disadvantages not disclosing |
| --- | --- | --- | --- | --- | --- | --- |
| #16 | No. Preferred to talk to someone in mother language.  Did not feel a need to talk to someone. | - Talked to a psychologist outside the university. - Did not experience stigma with the same age group but experienced stigma with older generations. - Is still trying to figure out how to talk about mental health. | Talking to a professional can help you gain good stuff. | Might experience stigma. | Not mentioned. | Not mentioned. |
| #22 | No. Did not had the right moment/person to talk to. | Talked to a psychologist outside the university. | Others can think along with you when things aren’t working out and how you can make things work again. | Taboo/ stigma on topic – stamp of having mental health issues. | People do not understand why you sometimes don’t respond to messages. | People won’t treat you different, and people cannot react in a disappointing way. |
| #13 | No. Not afraid of stigma.  Believes the university cannot help with mental health issues. | Psychologist outside the university, no need for talking within the university because of own support system and psychologist. | They already have some information about your mental health issues in case you will experience a very bad mental health phase or that you can create a safe space with a professional when you feel you need a room to cry or go when you are experiencing anxiety. | Not mentioned. | To prevent annoying responses from others and that they cannot help you in a wrong way which can make your mental health issues worse. | Not mentioned. |
| #51 | Disclosed to thesis supervisor. She felt she needed to explain why she was working more slowly and that she needed more rest. If she could do her thesis ‘normally’ she wouldn’t have told anyone. | Difficult to tell it but the supervisor responded in a good way for her, told her to rest and that it will be fine. | Might be that it is important to be honest and that you know that you are not the only one. | It will cost time and sees no use in it. | Not mentioned. | Was wondering if it would help if she would tell it. Deadlines and workload will remain the same. No added value in telling it to people. |
| Student 14 | Did not talk about it but her mentor was aware of the situation. Didn’t know with whom you can talk about mental health within UM.  Didn’t feel the need to share it. | - Taboo on topic. - Did not experience stigma. | Might be good if people know, in case you will miss a deadline or class. | Not mentioned. | That it stays with her. | Not mentioned. |
| Student 15 | No. Difficult to talk about it. | Not mentioned. | Don’t have to worry that she will miss an appointment. | - Afraid that people won’t take it seriously. - Feels like she cannot discuss it because of stigma. | Will not feel anxious (feels panic when she thinks she need to tell someone). But help might be good. |  |
| #17 | No. Does not know if there is help from the university with these kinds of complaints.  Enough social support – didn’t feel the need to disclose to other people | Believes there is stigma, especially in older generation – less in younger generation. | When you disclose this might help to solve your problems. | - Will cost a lot of energy to talk to someone. - Afraid of reactions when disclosing. - Disclosing will probably not change anything (e.g., rules regarding not joining a class). | Not mentioned. | Not disclosing will also not change anything. |
| #18 | No. Afraid of reactions when disclosing. | Experienced some stigma and racism. | Not mentioned. | Not mentioned. | You keep your privacy. | If you don’t disclose, complaints might get worse. |
| #24 | Disclosed to other students. Disclosed because someone else was also disclosing. | Didn’t experience stigma. But more difficult to tell parents compared to friends. | - Disclosing took a burden of her and likes to talk about it. - Talking helps and other people can help you. - Feeling more understood. - Other can explain what helped them. - Disclosing can help others to also disclose. | Not mentioned. | Not mentioned. | - People might treat you differently or might judge you. - When you don’t disclose and you have an emotional day or something, this can influence your work, and others don’t know why. |
| Student 25 | Disclosed to her mentor. She believes her mentor is someone who can guide her and that it is important for her study that someone knows about it, they can take it into account. | Not mentioned. | Maybe you can receive more support when you disclose. | - People don’t know how to deal with it when you disclose. - Experiences stigma. - Don’t want to burden others with her problems, don’t want to make them responsible. | Not mentioned. | Not mentioned. |

**Note**. In this framework matrix we mainly used the language as used by the participants.

**Framework matrix staff**

| Staff | Disclosed at UM (to whom) | Experience(s) | Advantages disclosing | Disadvantages disclosing | Advantages not disclosing | Disadvantages not disclosing |
| --- | --- | --- | --- | --- | --- | --- |
| #11 | Disclosed to supervisor and some other people. Doesn’t feel comfortable with not telling things and also couldn’t do all the work tasks and felt that she needed to disclose and had positive previous experiences with disclosing. | Received positive reactions and people were supportive. | - People can understand in which situation you are. - Receive support. | Might influence your career. | None. | None. |
| #21 | Disclosed to supervisors and to disability support and to colleagues when get to know them better.  They are the people you see on a weekly basis and felt like it could be told to them. Expected that they would be very understandful.  Doesn’t want to tell everyone because of stigma but felt like the need to say it because could probably not to all work activities e.g., some teaching activities or conferences. | Everybody has been very understanding and kind about it. | - When you disclose, they understand why you maybe don’t get all your work done. - Feeling (more) supported by supervisors. - Hoping for understanding and for more self-compassion. - Gained more freedom regarding working hours. - Adjustment to work activities. | People might act weird when you disclose. | - Social inclusion. - Avoiding stigma. | - That people think you are not able to do your work. - That people think you are not a member of the team if you don’t show up to anything – this can be avoided by disclosing. |
| #27 | First to other PhD Candidates (friends) later to colleagues, and also with supervisor/team and later with occupational physician.  Felt like she need to tell it because she couldn’t do her work properly but you also have a dependent relationship – is more difficult to talk with supervisors. |  | - A weight was lifted from her shoulders. - Noticed that other people also had issues and now they can support each other. - People were very understanding. - Receive social support. - Feeling relieved. - Time and work things can change. - That other people come to talk to you about your issues and that you are not the only one. - Better relationship with colleagues when you talk about these kinds of things, and you recognize things in each other. | - Stigma – especially self-administered stigma. - Difficult to admit that you cannot do your work as you should why everyone else seems like doing this. - Difficult to talk about it. - That other people come to you to talk about their issues and that is sometimes difficult. |  | - That you want to work again but that others say that it is better to take rest/work less. - Afraid that they will fire you. |
| #41 | No. Shares some things with people but would personally not define this as opening up because it is only small parts and not the core. |  | If you share about your mental health, you can explain things, for example if you cannot do a lot in a week. | Wants that people think that she is competent and skilled and doesn’t want to show weaknesses.  Might have influence on your career. |  |  |
| #52 | Talked with supervisor and colleagues. Felt the need to tell it because couldn’t work properly anymore | Good reaction from supervisor and this gave more strength to talk about it to colleagues. | - Supervisor did everything to help him. - Felt relieved. - Supervisor told him that they don’t judge him. - More beneficial than risky. - Cultural difference – people in the Netherlands are honest, you can talk about your mental health issues. - Disclosing can give you advantages. |  |  | - Did not experience disadvantages of disclosing. - Might think that there could be stigma. |
| #12 | Talked with supervisor, confidential PhD advisor, HR, and colleagues.  Because they would know what is possible in her situation, to offer guidance on sort of the legal aspects and they need to know what’s going on. She can work now but if it keeps going like this for years it might not and therefore, they should know.  You are a team, and you should discuss things and actions or decisions need to be taken and she feels like she had to tell. | - Good support. - Cultural difference makes it difficult to decide how much she can say. | - Not having to carry a burden alone – can feel alone and contributes to more mental distress. - Needed information and input and team can offer something – what she can or cannot do and what the possibilities are. - If you talk about it, it changes your perspective and ability to work with it. | - A fear that you don’t know how people will respond when you bring up something like this – didn’t feel like be judged for it but what is the implication. - Feels like a risk. - If you don’t talk about it, you are stuck in it. - You feel like you are complaining. - It can be hard to talk about it and not feel like you’re a drag on other people. - You get information you don’t want. |  |  |
| #23 | Talked with doctoral supervisory team.  Because previous experiences that it is better to tell it in your job and promotor was already aware of it (someone told promotor when it wasn’t going well).  Believes there is a stigma on mental health issues.  Already talked a lot about mental health issues which made it easier.  If they disapprove it, it is their problem. If they change their opinion after they know, he doesn’t want to work together anymore. | - Now positive experiences. - Previously bad experiences, people had an aversion to it. | - They promotor/supervisor) responded well on the issues. - It brought a lot. - Because they knew, they started working on it when it was necessary and benefited from that. - You give trust to the people who supervise you, better for trust relationship. - They can cooperate when they know, and you are sometimes not able to do some meetings. - Can support you. - Can think about solutions. - Less pressure.   People want to think together, want to make sure you can do your job and they care about you. | People can respond differently and negative e.g., don’t want to offer you a job. | Maybe they would be more restrained when offering certain tasks or responsibilities because they might think you cannot do this. |  |
| Staff #26 | Talked to students, to people she believes she can trust or would understand, to colleagues and to supervisor.  To show students that you can also be successful even when you have mental health issues. Was not really a choice because she was crying and felt like she needed to explain it.  Open about feelings and how she is doing.  Easier to talk with people who also have some difficult experiences in life. | Received good support. | - That people know that they are not the only one and that people know that they have they have a mental vulnerability but that it does not mean that they are strange or that they can never become something. - It clarifies things, you can talk about your tasks and if you are able to do everything. - Understanding from others - That you also can work less hours (on paper, talked with occupational physicist). - That you do not have to make excuses, that you just can say that you cannot do things. | - Misunderstanding and ability of other people to deal with emotions. - Uncertainty about what will this to do your career. - Afraid that it will work against you. - Stigma that people think that you cannot do your work properly if you have mental health issues or that they think you are scary. - Other people can be scared. - People can hurt you by their reaction. - You feel distance between people. - Expect that they would ask more often how you are doing but this did not happen. - That they will treat you very carefully and that they start to think about things you would need or make work adjustments you do not want. |  |  |
| #31 | Talked to other colleagues but, in their perception, not to one who could change something.   - Thinks when he would reach for help that they do not care about it. Less support and no willingness because of having a guest contract. Don’t dare to talk of feel silenced or no confidence in talking about it to people. - Talked when other people talked because they can share the struggle. - Because there is a shame on mental health. - Does not feel safe to talk about it. - Does not feel like the right thing to talk about something that is intimate to him. - People do not have the tools to help him. - Cultural thing to not talk about it. - Why burden others with your problem. - No social support to share. | - People are not interested or talk about other things. - Focus on other things because there is no money for support. - Received emotional support from people he talked to. | Talking about it could help make others also talk. | - Looking like a ranting overdramatic person. - Does not know how the department would deal with this. - You would not receive support. | - Peace in silencing himself. - To get on with the day without feeling that someone would approach you about it. - Not talking to fit in because otherwise you can break the harmony. | You do not resolve your issues. |
| #32 | To supervisor and colleague but not to colleagues who are very high ranked.   - Difficult because you might experience stigma but also because everyone has something, and you can help each other. - If you would talk to others about it, they can see that you can also do a lot with mental health issues, being a role model for students. - Easier to talk to women and younger people and people with same position or only a bit higher. - Circumstances can influence whether to talk about it. - Social environment advised to not talk about it because it might influence your career. - I am who I am. - Sometimes if there were issues, talked about it afterwards when she already found a solution. - Indirect stigma, stories from colleagues who experienced stigma. - Implicit stigma because not noticing social rules. - Work outcomes are still the same. |  | - That people would understand you better. - Relieves, it is clear. - People can listen to you. - If you are open about your issues, other people can also be open about it. | - Wondering if it would influence your career. - Misunderstanding from other people. |  |  |

**Note**. In this framework matrix we mainly used the language as used by the participants.

**Additional file 4: Students - preferences regarding DA**

|  | **Preferences towards content/form** | **How to be informed** | **When to use** |
| --- | --- | --- | --- |
| Student #16 | - Contact details (of people who can help you, e.g., through an introduction video). - Personal stories (including pictures). - Advantages of disclosing. - Practical information (about a consult). - Confidentiality (indicate that it will be confidential when you talk to someone). - User friendly (easy to use). - Brief information. - App or website (usable by phone). - Different forms of media (e.g., videos/diagrams [with what happens if you are not feeling well]). | - By university mails and updates. - Place the information in a mandatory course. - Mandatory appointment with university psychologist or mandatory form to fill out. | - At home (when you are alone and when you have time). |
| Student #22 | - List of advantages and outcomes of disclosing (e.g., can things be changed at the university if you tell). - Confidentiality (a website in which you anonymously can answer some questions regarding disclosing). - Personal stories (in video). - Website. - User-friendly (easy to use on the internet). - Brief information (text). - Media (chat to ask others’ questions). | - During a lecture (oral or show a video about the decision aid). - Poster. - Informed by mentor during mentor meeting. | - At home (maybe alone, maybe with parents). |
| Student #13 | - Contact details (of people who can help you including introductory pictures/videos) and automatic mails with contact details. - Advantages/disadvantages of disclosing (table). - Multiple choice questions/test/propositions to indicate that you have issues with something e.g., stress or school and that this will result in a match with a study advisor or psychologist. - App or website, preferably app. - Different forms of media (pictures and text). - Personal stories. - Chat (to ask general) questions). | - Brochures/posters with QR-code to use the app or referral to DA. - Spread the information at the UM (online). | - Should be accessible 24/7. - At home. |
| Student #51 | - Contact details (of people who can help you). - Advantages/disadvantages of disclosing (more attention to advantages). - Information that you are not the only one, that it is normal to ask for help. - Personal stories, from student but also of teachers who disclosed. - Questions (e.g, about age, study) – which results in with whom you could talk e.g., other students, teacher. - Meetings to talk about mental health with a group about a specific theme e.g., stress. - Online. - Videos. - Brief information (not too much text). - Numerical information about numbers regarding mental health. - Q&A or videos with fellow sufferers. | - Inform students during a class at the beginning of the year. - Via the Instagram ambassadors of different study programs. - Brochure/business card with a QR code to the DA and a nice quote and bright colours. | - In the beginning of the academic year. |
| Student #14 | - Contact details (of people who can help you including pictures). - Personal stories (short). - Show that it is okay to talk about your mental health issues. - Online tool. - Predispositions or questions (decisional tree) which results in with whom you can talk e.g., my mental health issues hinder my school performances. - Brief information (short and concise). - Graphics. - Chat (to talk with people about your issues or to decide if you should talk to someone). | - University website. - Brochure (e.g., in bag for first year students)/flyers. - During opening lectures of the course. | - At home. - At a moment when you have time for yourself. |
| Student #15 | - Contact details (of people who can help you). - Motivates you to talk, that you shouldn’t be afraid to talk. - Personal stories (experience stories of others, if the DA helped them, including pictures). - Information. - Online/social media. - Tables (e.g., to compare disadvantages or number about the amount of people who talk). - User friendly (easily accessible). - Chat to talk to someone. | - Beginning of the school year. - Mail. - On the learning management system used by the university. - During informational days. | - Beginning of the year, after Christmas Holiday. - At home. |
| Student #17 | - Contact details (of people who can help you). - Information about that others also suffer from mental health issues and that you shouldn’t feel weird if you have mental health issues. - Personal stories. - Quiz/questions. - Comparison looking for help/not and (dis)advantages. - Online, website. - Numbers about how many others suffer from mental health issues, examples. | - Posters. - Social media. - During lectures. | - At home. |
| Student #18 | - Contact details (of people who can help you). - Personal stories. - Advantages/disadvantages of disclosing (list). - Links to other websites which can help you. - Confidentiality (disclaimer that you can talk about it confidentially). - Online (usable on phone). - Question box /chat (e.g., during specific moments). - Brief information (not too much information). - User friendly (easily accessible). | - Website of the faculty. - Announcement. - Informed by Diversity & Inclusivity. - During tutorial. | - No preferences. |
| Student #24 | - Little introduction. - Tips about disclosing and to whom. - Personal stories. - Contact details (of people who can help you). - Frequently asked questions. - Chat (anonymous, also with other students). - Quiz (e.g., to figure out what is important to you). - Website (phone/laptop). - Social media (e.g., Instagram). - Advantages/disadvantages of disclosing (list). - Maybe short video (not too long). - Brief information (as little text as possible). - Media (clear and simple graphs and numbers). | - Social media. - Email. | - At home. |
| Student #25 | - Personal stories. - Information about how to talk to someone and how you can share it. - Meetings to talk with other people. - Advantages/disadvantages of disclosing. - Online. - Anonymous questionnaire. - Social media. - Personal stories (videos). - Pictures (also with a group of people who came together to talk about their mental health issues). - Brief information (short information and not too difficult). - Texting with a psychologist. | - Tutorial. - Lecture. - Social media. - WhatsApp group. | - At home. |

**Note**. In this framework matrix we mainly used the language as used by the participants.

**Additional file 5: Staff - preferences regarding DA**

|  | **Preferences towards content/form** | **How to be informed** | **When to use** |
| --- | --- | --- | --- |
| Staff #11 | - Information (e.g., that it is a strength that you can talk about your mental health issues, how to talk to your supervisor, positive consequences). - Movies. - Indicate that you can talk with others, also If you think that your problems aren’t important enough. - Advantages/disadvantages of disclosing (list). - Personal stories. - Chat (with psychologist/confidential person within UM/lived experience person). - Decisional tree. | - New employees can be informed via department head. - Existing UM communication channels. - Email. - Brochure. - Yearly updates. | - In a private situation. |
| Staff #21 | - Contact details (of people who can help you and guidance (step-by-step) in who you can talk to). - Ideas for opening up to line managers or a supervisor or for students a professor or thesis supervisor or colleagues. - Who could form a supportive network. - Advantages/disadvantages of disclosing (comparison and list). - Chat. - Website or poster. - Brief information (clear, structured, one page). - Text, graphics, and video (including subtitles). - Personal stories. | - Social media – especially to reach students. - UM internal media. - Emails from university. - Newsletters. - That people can go with you through the decision aid e.g., your supervisor. | - Own time – during lunch breaks, after/before/ during work. |
| Staff #27 | - Questions to answer if there is something going on. - Contact details (of people who can help you). - Advantages/disadvantages of disclosing (list). - Personal stories. - Frequently asked questions. - Prepositions – what is important to you. - Website or brochure. - Brief information (not too many details). - Different kind of information (e.g., mental health, physical health, graphics, numbers). - Personal stories. - Answering questions, resulting in a choice you could make. | - Mails. - Newsletters. - Via research line. | - No preferences. |
| Staff #41 | - Questionnaire which results in best option. - Advantages/disadvantages of disclosing (ranking which advantages suit you best). - Confidentiality (anonymous). - Personal stories. - Contact details (of people who can help you). - Chat/Ask the expert. - Information (e.g, how many people suffer from mental health issues, how to deal with people with mental health issues). - Website. - Brief information (not too much text, professional). | - Instagram. - Posters. - On the learning management system of the university. - Newsletter. - Email. | - On my own. |
| Staff #52 | - Contact details (of people who can help you). - Ask the expert. - Information about how many people (do not) talk about mental health. - Personal stories. - Chat function. - Advantages/disadvantages of disclosing (comparison). - Questionnaire (deciding whether to talk about your mental health issues, statements). - Brief information (not too much text and common language). - Media (e.g., interesting colours, words, drawings, visuals, graphs) - Online and visible. - Indicate that it is a free choice to (not) talk about your mental health issues. | - Sign/pamphlet to put on each desk of employees and walls. - Advertisements. - Emails. | - After office hours, weekend, lunch time. |
| Staf #12 | - Contact details (of people who can help you). - Information about the knowledge that pertains to the subject. - Personal stories. - Advantages/disadvantages of disclosing (visual representation, statements). - User friendly (easily accessible). - Website, flyer, in person or phone. - Help you to find out with whom you can talk best. | - Face to face. - Introduce to new students/employees. - By confidential advisor. - Make it visible e.g., in toilets, next to copy machine. - Posters. | - In private. |
| Staf #23 | - Information about what people might do with the information when you disclose to them. - A questionnaire or step-by-step plan. - Personal stories (video). - App. - User friendly (easy to use). - Confidentiality (information should be safe, privacy). - Forum. - A questionnaire resulting in advice or step-by-step plan about disclosing or not. | - By psychologists of the university. | - At home. |
| Staff #26 | - Information (e.g., about privacy, what the UM can offer, what happens when you disclose). - Advantages/disadvantages of disclosing. - Online tool/website. - Infographics. - Meeting with someone from HR to talk about (dis)advantages of disclosing. | - E-mails. - Posters. | - That you can use the decision aid with a person from the university e.g., a counsellor. |
| Staff #31 | - Information about where you can go. - Decisional tree. - Advantages/disadvantages of disclosing (in text). - Give some email address to ask contact details. - Information about confidentially. - Closed forum. - Online. - Brief information (straightforward). - Informational session to discuss it. | - Flyers. - Information booklet. - Information session. - Social media. | - When it’s convenient. |
| Staff #32 | - Information (e.g., about different options, other people who have mental health issues, numerical). - Contact details (of people who can help you). - Personal stories. - Online. - You click on options, and you will receive an answer about what options fits your situation. - Movies in which people show that they support that people talk about their mental health issues. | - Discuss existence of tool in yearly meetings with supervisor or with occupational physician. - Option to use the decision aid with a person e.g., confidential advisor. | - At home or with someone. |

**Note**. In this framework matrix we mainly used the language as used by the participants.
